# The golden hour of polymyxin B hemoperfusion in endotoxic shock: The basis for sequential extracorporeal therapy in sepsis

**DOI:** 10.1111/aor.13550

**Published:** 2019-09-02

**Authors:** Silvia De Rosa, Gianluca Villa, Claudio Ronco

**Affiliations:** ^1^ International Renal Research Institute of Vicenza Vicenza Italy; ^2^ Department of Anesthesiology and Intensive Care San Bortolo Hospital Vicenza Italy; ^3^ Department of Health Sciences, Section of Anaesthesiology, Intensive Care and Pain Medicine University of Florence Florence Italy; ^4^ Department of Nephrology, Dialysis and Transplantation and International Renal Research Institute of Vicenza, San Bortolo Hospital Vicenza Italy; ^5^ Department of Medicine University of Padova Padova Italy

**Keywords:** endotoxic shock, hemoperfusion, sequential extracorporeal therapy

## Abstract

Endotoxin is recognized as a major trigger of the immune response leading to pro‐ and anti‐inflammatory cytokine release, activation of the coagulation cascade, vasoplegic shock, and multiple organ dysfunction syndrome. A beneficial effect could be achieved through extracorporeal adsorption of circulating endotoxins in the blood as adjunctive treatment for unresponsive endotoxic shock. However, the precise clinical indication for its initiation is widely debated in the literature. Similar to the source control, microbiological cultures and antibiotics administration, endotoxin activity assay evaluation at regular intervals, and the targeted use of PMX‐B hemoperfusion could be lifesaving and adequate within the golden hour for the diagnosis and treatment of endotoxic shock.

Sepsis is the devastating result of a complex and dysregulated host response to an infection. It leads to an imbalance between pro‐ and anti‐inflammatory mechanisms and predisposes to the development of multiple organ dysfunction syndrome (MODS).^1^ Endotoxin has been recognized in most of the septic patients who developed MODS. Endotoxin is a major component of the cell wall of Gram‐negative bacteria, and it is recognized as a significant trigger of the immune response leading to pro‐ and anti‐inflammatory cytokine release, activation of the coagulation cascade, vasoplegic shock, and MODS. Endotoxic shock is an emergency condition where appropriate and prompt administration of antimicrobial therapy and proper source control may improve the patients' chance of survival. However, during bactericidal antimicrobial therapy, a clinically significant increase in circulating endotoxin levels might be observed due to bacterial lysis.^2^ Beyond the Gram‐negative sustained sepsis, endotoxin can also play a role in inflammatory bowel disease, ethanol‐induced liver disease, HIV infection, and in general in most of critically ill patients where endotoxin arises in the bloodstream by intestinal translocation due to altered gut permeability^3^ even in the absence of a positive blood culture to Gram‐negative infection. Due to bacterial lysis, endotoxin release may be different, late, or early, proportional to the number of killed pathogens, and further increase despite the decreased bacteremia.^4^

For this reason, a beneficial effect could be achieved through extracorporeal adsorption of circulating endotoxins in the blood as an adjunctive treatment for unresponsive endotoxic shock. Toraymyxin (Toray Medical Co., Ltd, Tokyo, Japan) is a cartridge containing polymyxin B (PMX‐B) immobilized on polystyrene fibers conceived for binding and neutralizing endotoxins from patients' blood through direct hemoperfusion. Beyond the pathophysiological rationale of the use of PMX‐B hemoperfusion (PMX‐B HP) for endotoxin removal, the precise clinical indication for its initiation is widely debated in the literature. According to the most updated evidence available from large randomized trials, septic shock patients with MODS score >9 and endotoxin activity assay (EAA) level ranging from 0.6 to 0.9 are those who may benefit the most from PMX‐B HP treatment in terms of improvement of survival.^5^, ^6^, ^7^ Although there is a little evidence supporting the efficacy of PMX‐B HP treatment in a selected population with endotoxic shock, the use of PMX‐B HP is challenged by a series of trials that show no benefit^8^ to support the routine use of PMX‐B HP to treat patients with sepsis or septic shock.

Apart from the general agreement to personalized indications for this complementary therapy, an appropriate timing indication for its initiation has not yet been proposed.^9^ Similar to continuous renal replacement therapy (CRRT) performed to counteract acute kidney injury (AKI), physicians should be aware of the effects that an untimely start of PMX‐B HP may have in patients with severe endotoxic shock, particularly for Gram‐negative sustained sepsis with severe bacteremia, where circulating endotoxin increases due to bactericidal antimicrobial therapy. In this condition, endotoxin neutralization by PMX‐B HP therapy might prevent or reduce the physiological (mainly hemodynamic) derangement typically observed during endotoxic shock. The underestimation of this concept could lead to a late, useless, and futile treatment. We believe that, similarly to the source control, microbiological cultures and antibiotics administration, EAA evaluation at regular intervals, and the targeted use of PMX‐B HP could be lifesaving and adequate within the golden hour for the diagnosis and treatment of endotoxic shock (Figure 1A). Also, we strongly suggest maintaining CRRT after treatment with PMX‐B HP when AKI concomitantly occurs, and renal replacement support is needed. In these conditions, highly adsorptive hemodiafilters can be considered. For instance, oXiris (AN69ST) is an AN69 membrane with PEI surface coating and immobilized heparin able to unselectively absorb endotoxin and pro‐ and anti‐inflammatory cytokines.^10^ In summary, the first therapeutic approach in unresponsive endotoxic shock is represented by PMX‐B HP, followed by targeted CRRT, as needed, and then scheduling the second session of PMX‐HP 18–24 hours after the first one (Figure 1A).

**Figure 1:**
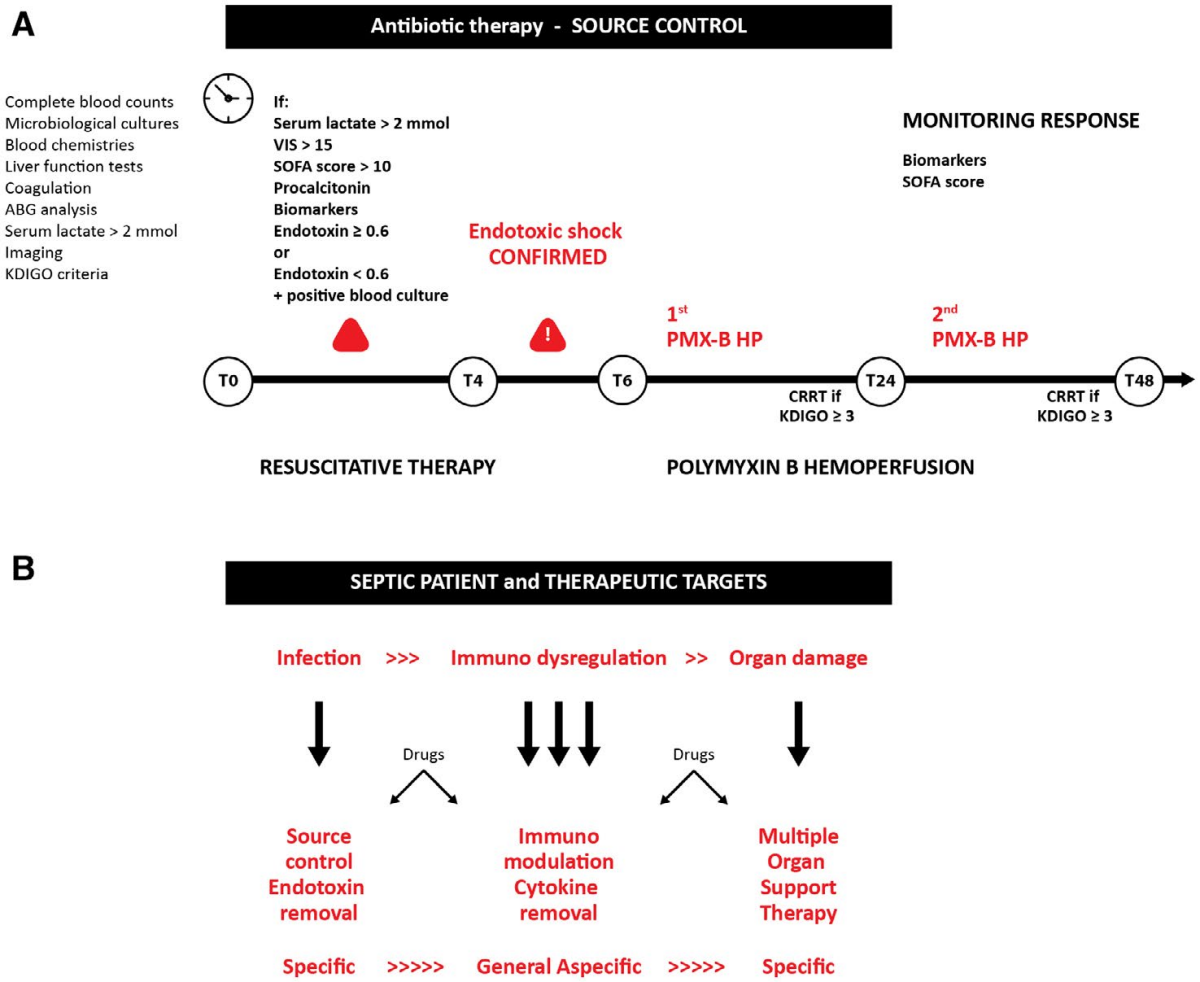
Flowchart for endotoxin shock management and therapeutic targets

According to our experience and clinical practice, the early phase of hemodynamic instability (vasoactive inotropic score [VIS] 10 < x < 30) during endotoxemia is usually associated with a slight decrease in renal function (kidney disease improving global outcomes [KDIGO] stages 1–2), biomarkers positivity and with an EAA level of 0.3 < x < 0.6. However, several concerns might be raised on the delayed approach of extracorporeal endotoxin removal in these situations. In particular, a rapid increase in endotoxin levels should be expected, particularly for patients with Gram‐negative sustained sepsis, due to endotoxin release during bactericidal treatment. Research and clinical protocols should consider an assessment of EAA at regular intervals to identify this rapid endotoxin increase.

Based on our experience, we strongly suggest to start extracorporeal endotoxin removal within 4 hours after source control and starting antibiotic therapy (Figure 1A). An over‐time assessment of EAA should be performed in these cases. The further and evident worsening of the renal function, as well as the increase in vasoactive support and a progressive increase in EAA, should corroborate the extracorporeal endotoxin removal initiation. However, an extracorporeal approach confined to patients with severe unresponsive shock (VIS >35 and sequential organ failure assessment score (SOFA) >15) and/or with a high level of EAA (higher than 0.9) should be carefully evaluated, particularly considering the potential futility of this expensive but efficient treatment if used improperly.

Three stages of a “golden hour” might be thus recognized in which the patient with a high risk of endotoxic shock might probably benefit most from endotoxin extracorporeal removal.

The timing becomes a crucial factor in the management of sepsis and the consequent MODS. Based on accurate monitoring and biological markers, an adjust prescription and appropriate delivery of what we defined sequential extracorporeal therapy in sepsis (SETS) might be guaranteed (Figure 1B).^9^ In the early phase, just after source control, PMX‐B HP can be the treatment of choice, followed by other extracorporeal blood purification therapies. When organ failure develops, extracorporeal therapies may become a broad spectrum support replacing or supporting the function of several organs such as heart, kidney, liver, and lungs. This is the rationale of extracorporeal organ support (ECOS), a new form of therapy in MODS.^11^

In a comprehensive approach to the endotoxic shock treatment, the evaluation of kidney function, vasopressor requirements, and EAA levels with source control and antibiotic therapy based on the time from sepsis diagnosis, may have a role to personalize the treatment for each specific patient. The dynamic monitoring and prescription could further refine the treatment personalization adequately responding to the criteria of precision medicine. Thus, not only a specific treatment could be provided for every single patient, but even a more specific treatment shall be provided for every moment that patient has a particular need during his/her ICU stay.

## ETHICAL APPROVAL AND CONSENT TO PARTICIPATE

This article does not contain any studies with human participants or animals performed by the author.

## CONFLICT OF INTEREST

The authors have no conflicts of interest to declare related to this manuscript.

## AUTHOR CONTRIBUTIONS

All authors contributed equally to the writing of this editorial. All authors read and approved the final manuscript.
